# Extensive Collection of Psychotropic Mushrooms with Determination of Their Tryptamine Alkaloids †

**DOI:** 10.3390/ijms232214068

**Published:** 2022-11-15

**Authors:** Klára Gotvaldová, Jan Borovička, Kateřina Hájková, Petra Cihlářová, Alan Rockefeller, Martin Kuchař

**Affiliations:** 1 Forensic Laboratory of Biologically Active Substances, Department of Chemistry of Natural Compounds, University of Chemistry and Technology Prague, Technická 5, 166 28 Praha 6—Dejvice, 166 28 Prague, Czech Republic; 2 Psychedelic Research Centre, National Institute of Mental Health, Topolová 748, 250 67 Klecany, Czech Republic; 3 Nuclear Physics Institute of the Czech Academy of Sciences, Hlavní 130, 250 68 Husinec-Řež, Czech Republic; 4 Institute of Geology of the Czech Academy of Sciences, Rozvojová 269, 165 00 Praha 6, 165 00 Prague, Czech Republic; 5 Mimosa Therapeutics, 1940 Union St., Oakland, CA 94607, USA

**Keywords:** psilocybin, psilocin, baeocystin, *Psilocybe*, hallucinogenic fungi

## Abstract

Since not only psilocybin (PSB) but also PSB-containing mushrooms are used for psychedelic therapy and microdosing, it is necessary to know their concentration variability in wild-grown mushrooms. This article aimed to determine the PSB, psilocin (PS), baeocystin (BA), norbaeocystin (NB), and aeruginascin (AE) concentrations in a large sample set of mushrooms belonging to genera previously reported to contain psychotropic tryptamines. Ultra-high performance liquid chromatography coupled with tandem mass spectrometry was used to quantify tryptamine alkaloids in the mushroom samples. Most mushroom collections were documented by fungarium specimens and/or ITS rDNA/LSU/EF1-α sequencing. Concentrations of five tryptamine alkaloids were determined in a large sample set of 226 fruiting bodies of 82 individual collections from seven mushroom genera. For many mushroom species, concentrations of BA, NB, and AE are reported for the first time. The highest PSB/PS concentrations were found in *Psilocybe* species, but no tryptamines were detected in the *P. fuscofulva* and *P. fimetaria* collections. The tryptamine concentrations in mushrooms are extremely variable, representing a problem for mushroom consumers due to the apparent risk of overdose. The varied cocktail of tryptamines in wild mushrooms could influence the medicinal effect compared to therapy with chemically pure PSB, posing a serious problem for data interpretation.

## 1. Introduction

**Figure 1:**
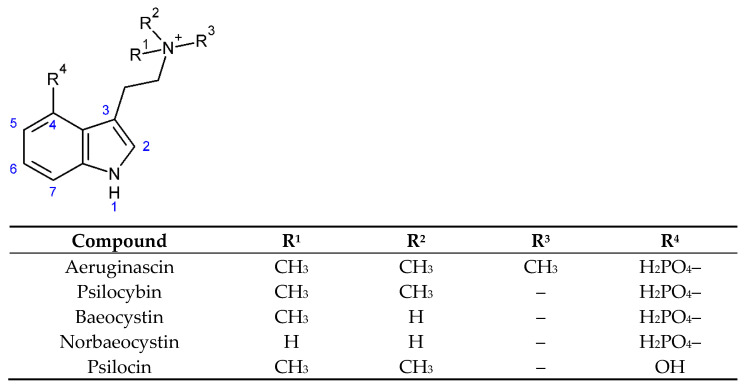
The structure of tryptamines quantified within this study.

Fungi are an understudied, biotechnologically valuable group of organisms [1]. Numerous biologically active compounds (secondary metabolites) have been described from mushrooms (macromycetes), and among them, psychotropic tryptamines have played fascinating roles in both ancient and modern human history. The main fungal tryptamines (Figure 1) are psilocybin (PSB) and psilocin (PS), while the minor tryptamines are baeocystin (BA), norbaeocystin (NB), and aeruginascin (AE) [2,3,4]. However, the psychotropic effect of BA, NB, and AE has not been fully established. It has been reported that the same dose of BA administered produced a psychotropic effect as the same dose of PSB [5]. However, BA and NB may not be psychoactive per se, but they can be transformed by PsiK kinase and PsiM methyltransferase to PS, which is psychotropic [4,6].

Interestingly, a recent investigation revealed that *Psilocybe* mushrooms also biosynthesize harmane, harmine, and a variety of other L-tryptophan-derived β-carbolines which are neuroactive compounds that serve as monoamine oxidase inhibitors (MAOI), which may potentiate the effect of tryptamines [7].

The capacity of psychotropic mushrooms to induce states of consciousness similar to mystical experiences could explain their widespread use in ritual and religious use [8]. Psychotropic mushrooms are distributed throughout the world and mostly belong to saprotrophic, but also ectomycorrhizal (symbiotic) genera: *Psilocybe* [9], *Panaeolus* including *Copelandia* [10], *Pluteus* [11], *Gymnopilus* [12], *Pholiotina* [13], *Galerina* [14], and *Inocybe* [15]. Curiously enough, PSB has also been recently reported from two taxa of entomopathogenic fungi [16].

For centuries and possibly for millennia, psychotropic mushrooms have been used as a sacrament in structured religious ceremonies and are still used as a “recreational drug” in the Western world [17,18]. However, PSB and PS are listed as *Schedule I* drugs under the *United Nations 1971 Convention on Psychotropic Substances* and the use of PSB/PS-containing mushrooms has been illegal in most countries since the 1960s and 1970s [19,20]. According to information from the literature [17] and public sources (e.g., Wikipedia page “Legal status of psilocybin mushrooms”), psilocybin-containing fungi are currently allowed to be consumed in Brazil, Jamaica, and the Netherlands. Compared to other legal and illegal drugs, psychotropic mushrooms represent a much lower risk to both users and society [21].

PSB is a serotonergic drug because it shares a common mechanism of action with serotonin, consisting of agonism in different serotonergic receptors, especially the 5-HT_2A_ receptors [22,23]. Serotonergic drugs produce profound modifications in perceptions and cognition [24]. There are promising results that indicate that PSB could be beneficial in psychedelic therapy (e.g., in the treatment of depression, addiction, post-traumatic stress, or mental disorders) [25,26,27,28].

Despite the fact that the taxonomy of psychotropic mushrooms is complicated, the characteristic bluing reaction makes them easier to identify in the field and “magic mushroom hunting” is a popular phenomenon, especially in Europe and North America including Mexico [29]. As recently demonstrated in *Psilocybe cubensis*, the enzymes laccase and phosphatase degrade PSB and initiate the bluing reaction [30]. The surface of the flesh and fruiting body of psychotropic mushrooms typically turns blue or blue-green when bruised or spontaneously [31,32]. Currently, our knowledge of the content of PSB and its derivates in the fruiting bodies of psychotropic mushrooms is insufficient. Most analytical studies on this subject date back before the 1990s when analytical possibilities were limited. In many articles, only qualitative PSB/PS results were published using simple chromatographic tests [12,33,34,35,36,37,38,39]. In more recent studies, quantitative data have been published on PSB/PS [40,41,42,43,44,45,46,47,48] and sometimes also on BA [10,49,50,51,52].

Quantitative data on AE and NB in mushrooms have only rarely been reported [13,50]. Furthermore, qualitative data on PSB/PS/BA content are available for only a limited number of mushroom species, and there is limited knowledge on the variations in the tryptamine concentrations in individual fruiting bodies among a single fungal species. Therefore, the aim of this study was to quantify both the major and minor fungal tryptamines in a broad sample set of psychotropic mushrooms by ultra-high performance liquid chromatography coupled with tandem mass spectrometry (UHPLC-MS/MS). Furthermore, we inspected variations in the tryptamine concentrations in a large sample set of the psychotropic *Psilocybe serbica* (and its varieties) from several sites in the Czech Republic.

## 2. Results and Discussion

The results of all of the measured mushroom species are summarized in Table 1. The detailed results of the individual samples are shown in heatmaps in Supplementary Figure S1. These results for whole fruiting bodies (cap and stipe) are given in mg/g dry mass. In total, 226 fruiting bodies of 82 individual collections of approx. 30 mushroom species were analyzed.

### 2.1. Analysis of Tryptamines in “Minor Psilocybin Genera” (Excl. Psilocybe)

In this section, non-*Psilocybe* species of the genera *Gymnopilus*, *Inocybe*, *Panaeolina*, *Panaeolus*, *Pholiotina*, and *Pluteus* were analyzed: 12 species, 23 collections, and 54 individual fruiting bodies (Table 1).

Among the genus *Gymnopilus*, relatively low concentrations of PS, PSB, and BA have been reported for *G. purpuratus* and *G. validipes*, with the highest level of 3.3 mg/g PSB [34,41,53]. We analyzed two fruiting bodies of our *G. dilepis* collection from Malaysia for which tryptamine data have never been published. When harvested, the mushrooms strongly turned blue-green but their tryptamine contents were lower than those formerly reported for *Gymnopilus* species, with the highest content of only 0.131 mg/g PSB and without detection of AE, NA, and NB.

Leaving aside *Boletus manicus*, which may contain hitherto unknown psychotropic indolic substances [54], *Inocybe* is the only ectomycorrhizal fungal genus reported to produce PSB and related tryptamine alkaloids. We analyzed three *Inocybe* species previously reported or suspected to be psychotropic, specifically *I. aeruginascens*, *I. calamistrata*, and *I. corydalina*. These mushrooms are often difficult to find, and we only had the opportunity to analyze the fungarium specimens. Analysis of such collections may be problematic due to the poor stability of tryptamine alkaloids [55].

In two fruiting bodies of *I*. *aeruginascens,* we quantified all of the monitored tryptamines except NB. Both samples contained similar concentrations of AE, PS, and PSB (0.280–0.283 mg/g AE, 0.005 mg/g PS, and 0.124–0.128 mg/g PSB), and the BA concentration was lower (0.038–0.039 mg/g). These results are comparable with Stijve et al. [56], but Gartz [50] reported circa 10× higher values for AE and PSB, and 100× higher values for BA. Such discrepancies may be attributed not only to analytical problems, but more likely to the age of the material analyzed in our laboratory.

**Figure 2:**
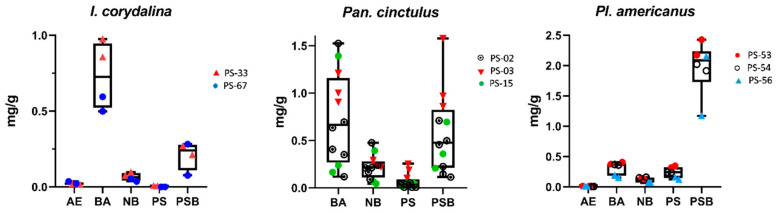
Tryptamine concentrations (mg/g in dry mass) in non-*Psilocybe* mushroom species are presented in boxplots for a minimum of two independent collections. The line in the boxplot represents the median and the whiskers signalize the minimum and maximum of the measured values. Tryptamine abbreviations: psilocybin (PSB), psilocin (PS), baeocystin (BA), norbaeocystin (NB), and aeruginascin (AE).

In *I*. *corydalina*, we analyzed four fruiting bodies of two individual fungarium collections (Figure 2). Curiously, the concentrations we found were even higher than those reported by Stijve and Kuyper [52], especially BA (0.499–0.975 mg/g) and 0.016–0.365 mg/g AE, 0.036–0.097 mg/g NB, less than or equal to 0.006 mg/g PS, and 0.076–0.282 mg/g PSB. It is interesting to compare the concentrations in PS-33 and PS-67, the latter analyzed nearly nine years after harvest. PS could not be detected in PS-67, but the concentrations of AE, BA, NB, and PSB were not remarkably lower than those in PS-33.

On the other hand, *I. calamistrata* used to be suspected of being psychotropic because of the blue-green tinge present at the stipe base (see, e.g., [57]), but PSB has not been found in the fruiting bodies [15,52,58]. Gartz [49] reported the occurrence of PSB, PS, and BA, but these data can be considered false-positive [59]. This study confirms the negative results of the previous studies.

*Panaeolina* and *Panaeolus* species (including taxa formerly classified as *Copelandia* or *Anellaria*) are commonly listed within the literature of psychotropic fungi ([29] and many others), but only a few species have been confirmed to contain psychotropic tryptamines. Despite the fact that a world monograph of *Panaeolus* exists [60], no molecular studies have been published to date on this genus and a critical revision relying on genetic markers is needed. Therefore, we have documented some of our collections by fungarium specimens and/or ITS rDNA sequences so that their determination can be corrected by future research.

*Panaeolus cinctulus* (referred to as *Panaeolus subbalteatus* in many previous studies) is known to contain psychotropic tryptamines. We analyzed 12 fruiting bodies from three different collections. Unfortunately, AE was not measured because these collections were processed before AE analysis was introduced into the method. These were quantified as 0.118–1.525 mg/g BA, 0.045–0.477 mg/g NB, 0.007–0.257 mg/g PS, and 0.114–1.578 mg/g PSB in *P*. *cinctulus*, which is shown in Figure 2. As pointed out by Stamets [61], tryptamine concentrations reported from this species are highly variable. Interestingly, Stijve and Kuyper [52] and Stijve and de Meier [62] found no PS, which was present in relatively low amounts in our samples.

In three additional taxa—*Panaeolus olivaceus*, *P. papilionaceus*, and *Panaeolina foenisecii*—none of the observed tryptamines were detected. In the latter two species, negative data were published earlier, but several positive detections of PS/PSB have also been published for *P. foenisecii*, which was reviewed by Allen and Merlin [63]. According to these authors, the positive results may be due to the misidentification of *P. foenisecii* with *P. cinctulus*.

A very interesting and rare species in our dataset is *Pholiotina cyanopus,* for which we analyzed three fruiting bodies from a single fungarium collection. *P*. *cyanopus* contained 0.821–1.360 mg/g BA, 0.247–0.565 mg/g NB, less than or equal to 0.062 mg/g PS, and less than or equal to 0.859 mg/g PSB. Our results were very similar to the previous study by Halama et al. [13], who also reported a trace concentration of AE.

Several *Pluteus* species from the section *Pluteus* are also known to contain psychotropic compounds [11]. We report the data for *P. salicinus*, which has already been analyzed for BA, PS, and PSB, and for *P*. *americanus* and *P*. *glaucotinctus,* which are supposed to be psychotropic due to their bluing reaction. Our data on BA, PS, and PSB in *P. salicinus* match those in previous studies [43,52], and concentrations of AE and NB (not yet reported) were generally low. An elevated NB content was observed in the single sample of *P. glaucotinctus*. In both *P. glaucotinctus* and *P. americanus* (and also *P. salicinus*), PSB was the major compound with concentrations exceeding 1 mg/g in most samples.

### 2.2. Quantification of Tryptamines in the Genus Psilocybe

**Figure 3:**
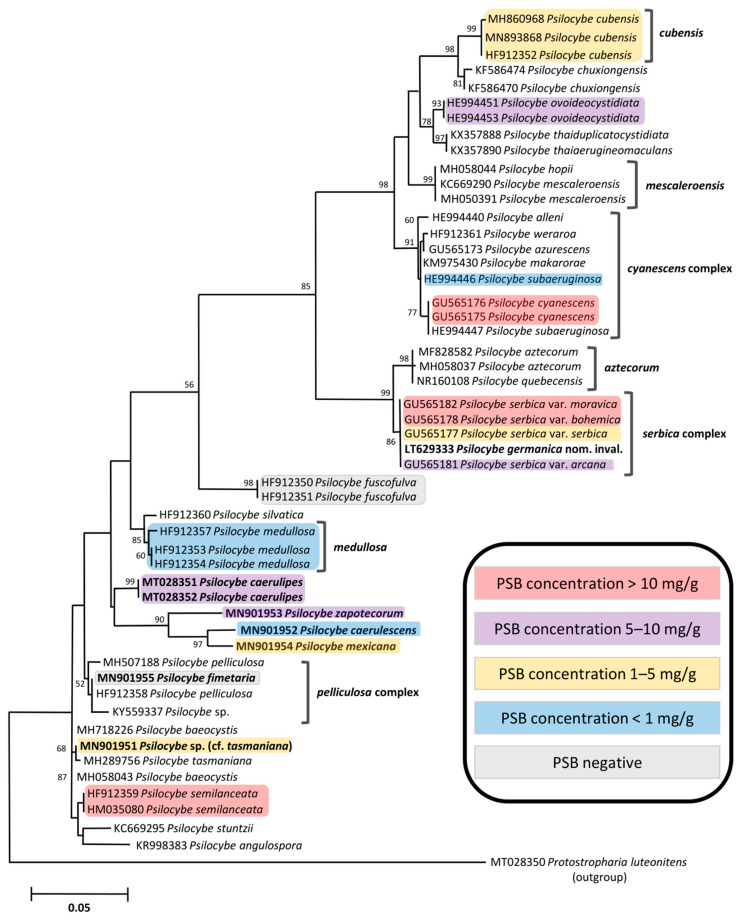
Phylogenetic placement of the studied *Psilocybe* species based on the ITS rDNA sequences (maximum likelihood tree computed in MEGA X). Bootstrap support values (>50, based on 1000 replicates) are presented along the branches. *Protostropharia luteonitens* was used as the outgroup to root the tree. The ITS rDNA sequences generated within this study are in bold. Information on psilocybin (PSB) concentrations is indicated.

The genus *Psilocybe* is the largest and the best-known group of psychotropic mushrooms. We analyzed 87 fruiting bodies from 31 individual collections of 14 *Psilocybe* species. The phylogenetic tree (ITS rDNA) of the genus is presented in Figure 3, where the maximum PSB concentrations are also indicated; the phylogenetic position of all analyzed species is indicated in the tree. Here, we comment on the results for the *Psilocybe* species from the top to the bottom of the tree, but not on the species complex of *P. serbica*, which will be discussed separately.

**Figure 4:**
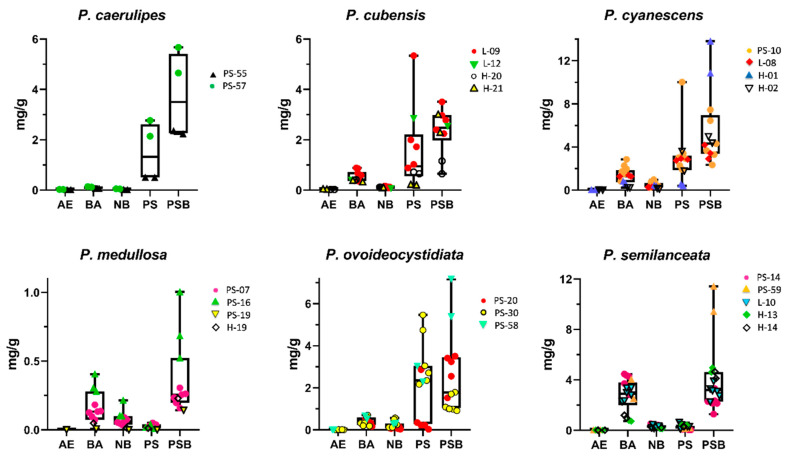
Tryptamine concentrations (mg/g in dry mass) in six *Psilocybe* species presented in boxplots for a minimum of two individual collections. The line in the boxplot represents the median and the whiskers signalize the minimum and maximum of the measured values.

*P. cubensis* is the most frequently cultivated psychotropic mushroom in the world, and it produces large fruiting bodies. We analyzed nine fruiting bodies from four individual collections, where we quantified 0.026–0.053 mg/g AE, 0.139–0.881 mg/g BA, 0.044–0.161 mg/g NB, 0.208–5.344 mg/g PS, and 0.651–3.509 mg/g PSB. Concentrations of BA, PS, and PSB corresponded to the tryptamine ranges from previous studies [3,27,41], and similar NB and AG concentrations were reported in our previous study [55]. *Psilocybe ovoideocystidiata* is rather closely related to *P. cubensis* (Figure 3). The tryptamine concentration ranges determined in eight fruiting bodies from three collections were similar to *P. cubensis* (Table 1, Figure 4) and higher than those reported by Allen et al. [40].

The taxa belonging to the *P. cyanescens* complex only showed subtle differences in the ITS rDNA marker, as can be seen in the tree (Figure 3). We only analyzed two species of this complex: *P. cyanescens* and *P. subaeruginosa*. *Psilocybe cyanescens* is known as the “Wavy cap” on the Pacific Coast of the USA and is probably the best-known wood-rotting species growing in the USA. This mushroom is not indigenous to Europe where it was introduced in the 20th century [31]. *P. cyanescens* is known for its high BA, PS, and PSB concentrations [3,41,45,52]. Our results for four European collections analyzed in this study confirmed that *P. cyanescens* is one of the most “potent” species: 0.011–0.039 mg/g AE, 0.216–2.852 mg/g BA, 0.102–0.978 mg/g NB, 0.409–10.018 mg/g PS, and 2.340–13.808 mg/g PSB. Literature data for PS and PSB in *P. subaeruginosa* are based on methods using older detection techniques, namely, the UV detector and fluorescence detector [64] and chemiluminescence [65], where the reported PSB range was 0.0–2.1 mg/g and the PS content was only 0.038 mg/g [65]. Our results for all tryptamines in *P. subaeruginosa* were very low (the highest of 0.33 mg/g PS) and did not correspond much to those found in other species of the *P. cyanescens* complex. We speculate that this species may produce much higher tryptamine concentrations and additional analyses are therefore desired.

Tryptamines were not detected in *Psilocybe fuscofulva* (*P. atrobrunnea* auct., *P. turficola* nom. inval.). This species is present in an isolated lineage in the tree (Figure 3) and does not turn blue/green when bruised. Our negative results for this species confirm those of the previous studies [66,67] and correspond with the molecular evidence provided by Reynolds et al. [68]. *P. fuscofulva* is the only non-psychotropic member of the genus *Psilocybe* in the modern concept [69]. However, lower in the tree, another tryptamine-negative species can be seen: *Psilocybe fimetaria*.

Although one of the *P. fimetaria* collections was analyzed only a few months after sampling (PS-65), we were unable to detect any of the analyzed tryptamines. This collection (PRM 951396) was found in the Czech Republic and was primarily determined according to the morphological characters. The phylogenetic analysis shows its affinity to *P. pelliculosa* (Figure 3), a psychotropic species known from the USA [29]. One of the ITS rDNA sequences of *P. pelliculosa* was even 100% similar to that of *P. fimetaria*, but their EF1-a gene sequences are apparently distinct (the sequences HF912341 and LR760712 show a match of only 94%). This would suggest that the ITS rDNA molecular marker is not always a reliable character for species delimitation in *Psilocybe* (which is also indicated in the *Psilocybe cyanescens* complex). According to the literature [70,71], *Psilocybe fimetaria* turns blue when bruised, however, the bluing reaction was not observed in the Czech collection. Because no other sequences of *P. fimetaria* are available in public databases, we were unable to discuss the taxonomic identity of our collection. The absence of tryptamine alkaloids in our samples could be explained, for example, by (i) low initial concentration and following degradation before analysis, and (ii) an existence of two *Psilocybe* species corresponding to the morphological concept of *P. fimetaria*, one psychotropic and one non-psychotropic, as not only the bluing reaction but also PSB was reported in *P. fimetaria* [33]. Therefore, a thorough analysis and multi-gene sequencing of additional collections of *P. fimetaria* are needed.

*Psilocybe medullosa* is a rare European species that does not turn blue when bruised but the chemical tests indicated an occurrence of PSB and PS at low levels [67]. We analyzed 11 fruiting bodies from three different collections and measured no AE, 0.007–0.406 mg/g BA, 0.000–0.215 mg/g NB, 0.000–0.051 mg/g PS, and 0.143–1.003 mg/g PSB. The tryptamine concentrations were rather low, but certainly not negligible.

*Psilocybe caerulipes* is widespread but not common in the USA, with no available analytical data on tryptamines [29]. Our results for the two collections showed relatively high concentrations: 0.018–0.028 mg/g AE, 0.63–0.141 mg/g BA, 0.019–0.052 mg/g NB, 0.501–2.770 mg/g PS, and 2.234–5.674 mg/g PSB.

Furthermore, we had the opportunity to analyze three well-known Mexican *Psilocybe* species: *P. caerulescens*, *P. mexicana*, and *P. zapotecorum* (for current taxonomic concepts of these species see [72]). Due to the lack of material (two fruiting bodies per collection), no fungarium material was deposited, but all collections were documented by ITS rDNA sequencing. The lowest tryptamine content was found in *P. caerulescens* with the highest concentration of only 0.413 mg/g PS. Higher concentrations were found in *P. mexicana,* from which PS and PSB were isolated in 1958 by Albert Hofmann [73]. We quantified traces of AE, 0.254–0.324 mg/g BA, 0.159–0.203 mg/g NB, 1.944–1.974 mg/g PS, and 3.286–3.934 mg/g PSB in these mushrooms which is consistent with the historical findings by Hofmann et al. [74]. When compared to *P. mexicana*, in *P. zapotecorum*, we found a similar NB content, but lower PS (0.293–0.367 mg/g) and considerably higher PSB (9.022–9.653 mg/g). To the best of our knowledge, these are the first analytical data for tryptamines in *P. zapotecorum*.

One undetermined *Psilocybe* species donated from Australia appeared to have the same ITS rDNA sequence as one labeled as *P. tasmaniana* (GenBank MH289756), but the spore size of our collection did not match that reported for this species [70]; we therefore considered this collection *Psilocybe* cf. *tasmaniana*. Tryptamine measurements revealed relatively low concentrations, which are published for the first time for this species: 0.282–0.965 mg/g BA, 0.031–0.144 mg/g NB, 1.489–2.051 mg/g PS, and 0.512–1.892 mg/g PSB; AE was not analyzed because the method was not available.

Last but not least, grassland inhabiting *P. semilanceata* (Liberty Cap) was analyzed as one of the most well-known and widespread temperate species. There is rich analytical data on tryptamines in this species [3,41,42,43,45,46,52], but our quantification of AE and NB is novel. Relatively high BA, high PSB, and low PS concentrations are typical for this species, which is consistent with our measurements (Figure 4).

### 2.3. Quantification of Tryptamines in the Psilocybe serbica Complex

The *Psilocybe serbica* complex contains several taxa formerly classified at the species level [31]. However, phylogenetic analysis based on three molecular markers did not prove a significant difference between *P. serbica*, *P. bohemica*, *P. arcana*, *P. moravica*, and *P. moravica* var. *sternberkiana*. Therefore, all taxa are now considered conspecific with *P. serbica* or may be treated as varieties [67,71,75]. The phylogenetic analysis (Figure 3) also revealed that the invalidly described “*Psilocybe germanica*” (Gartz & Wiedemann 2015, Drug Testing and Analysis 7: 853–857) did not differ from the validly described taxa of the *Psilocybe serbica* complex (however, no material was available for tryptamine analysis).

**Figure 5:**
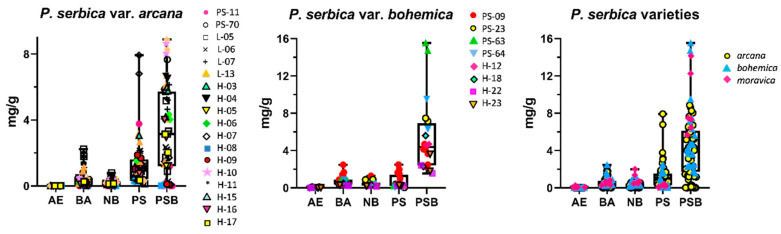
Tryptamine concentrations (mg/g in dry mass) in *Psilocybe serbica* var. *bohemica*, *Psilocybe serbica* var. *arcana* and the summarized data for all *Psilocybe serbica* varieties under study are presented in boxplots. The line in the boxplot represents the median and the whiskers signalize the minimum and maximum of the measured values.

*Psilocybe serbica* and its varieties are very common in certain regions of the Czech Republic, which enabled us to analyze a rich sample population, particularly of var. *arcana* and var. *bohemica*; one collection could not be attributed to a specific variety and is indicated as *P. serbica* in the dataset. Unfortunately, the tryptamine concentration data presented here (Table 1, Figure 5) cannot be accurately compared with earlier data published in the literature because the particular varieties were not well-described or distinguished in the past.

The most abundant taxon we studied from this complex was var. *arcana*, where we analyzed 54 fruiting bodies from 18 individual collections. Our dataset is the most extensive out of all published studies. We measured 0.000–0.024 mg/g AE, 0.000–2.237 mg/g BA, 0.000–0.794 mg/g NB, 0.412–7.922 mg/g PS, and 0.002–8.878 mg/g PSB. The concentrations are highly variable, which was also observed in var. *bohemica*. Curiously enough, the concentrations of PS (0.027–2.485 mg/g) and PSB (1.553–15.543 mg/g) determined in var. *bohemica* (20 fruiting bodies, eight collections) were significantly higher (*t*-test, *p* < 0.05) than those in var. *arcana*; the concentrations of AE, BA, and NB were roughly similar. The concentration of 15.543 mg/g PSB in var. *bohemica* was the highest determined in the whole dataset and *P. serbica* var. *bohemica* is therefore comparable with the highly potent *P. azurescens* with PSB concentrations reported of up to 17.8 mg/g PSB [58].

Previous studies [3,52,53] referring to var. *bohemica* have reported similar or slightly lower tryptamine levels—in light of our findings, this could be caused by including var. *arcana* in the dataset. On the other hand, Stříbrný et al. [45], who distinguished between var. *bohemica* and var. *arcana*, did not observe such a difference in the PS/PSB content in these varieties and PS was significantly higher than PSB in var. *bohemica*. This discrepancy could be attributed to numerous factors, especially a low sample population (only one collection per variety was analyzed). *Psilocybe serbica* var. *moravica* is rare and only six fruiting bodies of one collection could be analyzed. The tryptamine concentrations were similar to those of var. *bohemica*, where low PS (0.061–0.386 mg/g) and high PSB (5.655–14.158 mg/g) concentrations were observed.

Despite the fact that the morphological differences between var. *arcana* and var. *bohemica* have not been confirmed by molecular studies, there is a significant difference in their PS and PSB composition. However, this result should be taken with care, and a more detailed study focused on this difference should carefully verify this observation. Because of the insufficient sample population, we were not able to compare the PS and PSB concentrations in var. *moravica*; the available data suggest a similarity to var. *bohemica*. We also cannot explain the striking variation in the tryptamine concentrations observed in samples probably originating from the same mycelium, and possible factors that influence this phenomenon, both environmental and biological, should be investigated in the future.

### 2.4. Remarks

The large variability in the concentrations of tryptamines in fruiting bodies observed in this study deserves more attention. According to our data, some *Psilocybe* species possibly contain higher concentrations of tryptamines than others (species-dependent). The differences between species might correspond to the activity of enzymes (PsiD, PsiH, PsiK, PsiM) that catalyze the biosynthesis of NB, BA, PSB, and PS [4,76].

However, the development stages of fruit-bodies and the climatic conditions may also represent important factors that regulate the production of psychotropic tryptamines [77]. These are also hydrophilic substances [78], so we did not exclude the possibility that they could be leached from the fruiting bodies into the surrounding environment by rainwater. Furthermore, the concentration of tryptamines may depend on the substrate composition: the content and bioavailability of nitrogen, phosphate, and the amino acid tryptophan, which serves as a precursor for the biosynthesis of PSB and similar tryptamines [77,79]. Tryptophan can be enriched in the substrate by fertilizing the soil with decomposed plants or animal dung [80], so fruiting bodies harvested, e.g., from woodchip beds might contain higher tryptamine concentrations than those from natural sites. However, the actual influence of such factors has yet to be investigated. Last but not least, the concentrations may be artificially affected by sample processing and storage before analysis [55].

According to our experience during this study, there is great scientific and public interest in the concentrations of tryptamine alkaloids in psychotropic mushrooms. However, their illegal status greatly complicates their taxonomic and chemical investigation. Donating PS/PSB containing mushroom samples for scientific study is associated with the risk of criminal prosecution, but without such assistance, our study would be limited to the mushroom species growing in the Czech Republic, where the work of the analytical team was within the legal boundaries.

Furthermore, the poor stability of tryptamines complicates the evaluation of the data [55], especially when the mushrooms were harvested by volunteers with limited laboratory equipment and experience—it is essentially not possible to ensure completely identical processing of the samples prior to analysis. The delay between collecting and analysis also affects the analytical results to some extent, but the relatively high tryptamine concentrations in old fungarium samples (e.g., *Inocybe corydalina* PS-67) suggest that the degradation of tryptamines in dried fruit-bodies is not as rapid as in the pulverized samples investigated by Gotvaldová et al. [55].

In addition, some psychotropic mushrooms are difficult to find in the field (or difficult to find in sufficient quantities), so only old fungarium collections were available for analysis. In any case, we tried to ensure and present the most interesting composition of psychotropic mushrooms available with the quality corresponding the analytical results.

## 3. Materials and Methods

### 3.1. Chemicals and Standards

Methanol LC-MS CHROMASOL™ and eluent additives for UHPLC-MS/MS—acetic acid, formic acid, and ammonium formate—were obtained from Honeywell, Fluka™, Charlotte, NC, USA. Ultrapure water, 18.2 MΩ·cm, was produced by the Smart2Pure 12 UV system (Thermo Fisher Scientific—Barnstead, Germany). The analytical standards including PSB and PS were purchased from Cayman Chemical, Ann Arbor, MI, USA. The reference standards of AE, BA, and NB were synthesized at the Forensic Laboratory of Biologically Active Substances at UCT Prague according to our previous work [55].

### 3.2. Collection of Mushroom Samples

A major part of the mushroom samples was obtained by the authors of this study and their co-workers. These were collected in their natural habitats in the Czech Republic and Malaysia. Furthermore, mushroom collections were provided in response to requests on social media by anonymous donors from Australia, Bosnia and Herzegovina, France, Germany, Italy, Mexico, Sweden, and the USA. Some mushroom samples (mainly *Inocybe* spp.) were donated for analysis from the fungarium of the Mycological Department of the National Museum, Prague (PRM) and private fungaria.

The collectors were instructed to dry the mushrooms gently in the air (near a heater or in an air dryer at no more than about 40 °C) and promptly sent them to the Forensic Laboratory of Biologically Active Substances in Prague, which is authorized to handle illegal psychotropic substances. Dried fungal samples were stored in the dark at 20 °C until analysis. The time between mushroom collection and analysis is shown in Supplementary Table S1. Mushroom species were identified by Jan Borovička according to the current taxonomy [32,67,71,75,81,82] using micromorphological and/or molecular characters (DNA sequencing, see below). When possible, representative parts of the mushroom collections were deposited in the fungarium of the Mycological Department, National Museum, Prague, Czech Republic (PRM). Wild *Stropharia aeruginosa* and cultivated *Agaricus bisporus* (sold on the market) were used as mushroom negative controls in the tryptamine measurements. For the list of analyzed mushroom species and further details on the collections (collection date, geographic origin, etc.) see Supplementary Table S1. For the list of complete scientific mycological names of mushroom species including the authors, see Supplementary Table S2.

### 3.3. Extraction

The dried fruiting bodies were homogenized by a ceramic mortar and pestle to a fine powder. To extract the sample, fungal powder (10.0 ± 0.5 mg) was mixed with 1.0 mL of 0.5% acetic acid (*v*/*v*) in methanol. The mixtures were shaken by a vortex at 13× *g* at 20 °C for one hour. In the next step, the testing tubes were centrifuged for 10 min at 857× *g* at 20 °C. These suspensions were filtered using Eppendorf tubes with a 0.2 µm PTFE microfilter (Ciro, Hudson, WI, USA) in a centrifuge (15,256× *g*, 5 min, 20 °C). Then, re-extractions of the remaining pellets were carried out with 1 mL methanol and vortexed and ultrafiltrated using the same conditions as described above. To prevent degradation of the analyzed alkaloids, the extraction solvents were purged with a stream of nitrogen, and the sets of testing tubes were covered with aluminum foil.

Mixtures of 100 µL of the final supernatant and 900 µL solvent of 10 mM ammonium formate with 0.1% formic acid in methanol/water (1/9, *v*/*v*) stored in brown glass vials (Assistent-Glaswarenfabrik Karl Hecht, Sondheim vor der Rhön, Germany) were used for analysis using UHPLC-MS/MS. Consequently, these samples were further diluted (10×, 100×, and 1000×) due to different concentrations of analytes.

### 3.4. Instrumental Analysis

The separation and quantification of the five analyzed tryptamines AE, BA, NB, PS, and PSB in mushroom biomass was carried using the UHPLC 1290 Infinity assembly (Agilent Technologies, Santa Clara, CA, USA) with a Zorbax Eclipse Plus C18 column, 100 × 2.1 mm, 1.8 µm and with a Zorbax Eclipse Plus C18, 5 × 2.1 mm, 1.8 µm pre-column (Agilent Technologies, Santa Clara, CA, USA) coupled with a triple quadrupole 6460 spectrometer (Agilent Technologies, Santa Clara, CA, USA). This method was used based on a previous publication (Gotvaldová et al., 2021 [55]). Column separation was performed at 40 °C. In the method, mobile phase A—10 mM ammonium formate with 0.1% (*v*/*v*) formic acid—and mobile phase B—10 mM ammonium formate with 0.1% (*v*/*v*) formic acid in methanol—were used. The gradient elution was used, the flow rate was 0.25 mL/min, and the injection volume of the sample was 3 μL.

In the MS system, an electrospray ionization in positive mode (ESI^+^) was used for all analytes. Optimal MS operating conditions were set as follows: drying gas temperature (nitrogen) of 340 °C, drying gas flow of 10 L/min, nebulizer (nitrogen) of 25 psig, sheath gas temperature (nitrogen) of 400 °C, sheath gas flow of 12 L/min, and capillary voltage of 2300 V. The dynamic multiple reaction monitoring (dMRM) acquisition mode was utilized for analysis. For all analytes except PS, the two most intensive transitions were used, whereas for PS, three transitions were used. The detection limit (LOD) was 1.0 ng/mL for NB and 0.1 ng/mL for other analytes. The quantification limit (LOQ) was 5.0 ng/mL for NB and 0.5 ng/mL for the other analytes. The total analysis time was 7 min.

### 3.5. Molecular Methods and Phylogeny

In order to determine or document some of the studied mushroom collections, automated DNA sequencing of the ITS rDNA, LSU, and EF1-α molecular markers was performed at Macrogen Europe. Genomic DNA was extracted from dried fruiting bodies using either the NucleoSpin Plant II DNA Isolation Kit (Macherey-Nagel) or the NaOH extraction protocol [83]. For details on the DNA extraction, PCR conditions, primers, PCR product purification, and sequencing, see [75]. The sequences were submitted to the GenBank/EMBL-Bank databases under the accession numbers MN901948-MN901955, MN900720, MW352021-MW352022, MT028350, and LR760712. For the list of sequenced collections, see Supplementary Table S1.

For the phylogenetic analysis (ITS rDNA), reliable *Psilocybe* sequences were retrieved from the GenBank database, mostly based on taxonomic papers on this genus [67,75,82,84,85,86,87,88,89], but also unpublished sequences that the authors believed to be correctly identified (direct submissions to GenBank, with no available reference). Sequences of insufficient length were omitted. The sequences were aligned with MAFFT online version 7 [90] using the E-INS-i strategy with default settings. The poorly aligned positions in the alignment were removed with Gblocks v. 0.91b [91] with options for a less stringent selection. The evolutionary history was inferred by MEGA X using the maximum likelihood (ML) method based on the Kimura 2-parameter model [92], which was the best-fitting model according to the model test analysis in MEGA X. The tree with the highest log-likelihood (−2513.31) is shown. The percentage of trees in which the associated taxa clustered together is shown next to the branches (maximum likelihood bootstrap values). The tree is drawn to scale, with branch lengths measured in the number of substitutions per site. The analysis involved 52 nucleotide sequences and a total of 588 positions were in the final dataset, of which 147 were variable and 35 were singletons. *Stropharia luteonitens* (MT028350) was used as the outgroup.

## 4. Conclusions

Mushroom fruiting bodies of the genera *Gymnopilus*, *Inocybe*, *Panaeolus*, *Pluteus*, and *Psilocybe* may contain high concentrations of tryptamine alkaloids. In this study, we measured the concentrations of AE, BA, NB, PS, and PSB for which quantitative data on AE and NB in particular are reported for the first time in the literature. A large variation in the concentrations of all tryptamines was observed for many mushroom species and possible factors that influence this variability should be studied in the future.

The highest concentrations of PS/PSB were found in *Psilocybe* species. The highest content of 15.54 mg/g PSB was found in *Psilocybe serbica* var. *bohemica*. Interestingly, a significant difference was observed in the PS and PSB concentrations in *P. serbica* var. *bohemica* and *P. serbica* var. *arcana*. This indicates a possible chemotaxonomic significance of PS/PSB concentrations in the *P. serbica* complex and should be thoroughly investigated in further studies. However, there are species such as *P. medullosa* that probably contain relatively low PS/PSB concentrations, and no tryptamines were detected in *P. fuscofulva* and *P. fimetaria*. The concentrations of tryptamines seem to be species-dependent in the genus *Psilocybe*, which is a phenomenon not only observed for the secondary metabolites, but also for trace element accumulation ability in many mushroom genera [93,94].

The variations in the PS/PSB concentrations in mushrooms pose a potential risk to consumers as they make it difficult to estimate the appropriate dose of the drug. Furthermore, the variable cocktail of other tryptamine alkaloids in mushroom species detected in this and other studies [7] may influence their psychotropic effect in humans. This could be of interest to researchers investigating their potential use in psychotherapy because the psychotropic effect of the full spectrum of natural mushrooms may differ from that of pharmaceutically pure PS/PSB. Therefore, more studies are needed on the concentrations of minor tryptamine alkaloids in psychotropic mushrooms.

## Figures and Tables

**Table 1:** Concentrations of tryptamine alkaloids (mg/g in dry mass) in the investigated mushroom species. Aeruginascin (AE), baeocystin (BA), norbaeocystin (NB), psilocin (PS), and psilocybin (PSB).

Mushroom Species	fb	n	AE	BA	NB	PS	PSB
*Gymnopilus dilepis*	2	1	<LOD	0.002–0.005	<LOD	0.024–0.063	0.031–0.131
*Inocybe aeruginascens*	2	1	0.280–0.283	0.038–0.039	<LOD	0.005	0.124–0.128
*Inocybe calamistrata*	9	5	<LOD	<LOD	<LOD	<LOD	<LOD
*Inocybe corydalina*	4	2	0.016–0.365	0.499–0.975	0.036–0.097	0.000–0.006	0.076–0.282
*Panaeolus cinctulus*	12	3	―	0.118–1.525	0.045–0.477	0.007–0.257	0.114–1.578
*Panaeolus foenisecii*	8	2	<LOD	<LOD	<LOD	<LOD	<LOD
*Panaeolus olivaceus*	2	1	―	<LOD	<LOD	<LOD	<LOD
*Panaeolus papilionaceus*	2	1	―	<LOD	<LOD	<LOD	<LOD
*Pholiotina cyanopus*	3	1	―	0.821–1.360	0.247–0.565	0.000–0.062	0.000–0.859
*Pluteus americanus*	6	3	0.008–0.014	0.154–0.410	0.067–0.162	0.123–0.347	1.172–2.428
*Pluteus glaucotinctus*	1	1	0.012	0.224	0.484	0.013	1.939
*Pluteus salicinus*	3	2	0.014–0.024	0.032–0.157	0.018–0.044	0.037–0.070	0.306–1.353
*Psilocybe caerulescens*	2	1	<LOQ	0.009–0.013	<LOD	0.341–0.413	0.225–0.310
*Psilocybe caerulipes*	4	2	0.018–0.028	0.063–0.141	0.019–0.052	0.501–2.770	2.234–5.674
*Psilocybe cubensis*	9	4	0.026–0.053	0.139–0.881	0.044–0.161	0.208–5.344	0.651–3.509
*Psilocybe cyanescens*	13	4	0.011–0.039	0.216–2.852	0.102–0.978	0.409–10.018	2.340–13.808
*Psilocybe fimetaria*	4	2	<LOD	<LOD	<LOD	<LOD	<LOD
*Psilocybe fuscofulva*	6	3	<LOD	<LOD	<LOD	<LOD	<LOD
*Psilocybe medullosa*	11	3	<LOD	0.007–0.406	0.000–0.215	0.000–0.051	0.143–1.003
*Psilocybe mexicana*	2	1	0.006–0.007	0.254–0.328	0.159–0.203	1.944–1.974	3.286–3.934
*Psilocybe ovoideocystidiata*	8	3	0.004–0.013	0.164–0.699	0.031–0.568	0.026–5.464	0.914–7.172
*Psilocybe semilanceata*	18	5	0.010–0.033	0.725–4.467	0.121–0.510	0.033–0.619	1.280–11.421
*Psilocybe serbica*	5	1	―	1.748–3.385	1.010–1.946	0.215–3.810	1.562–3.957
*Psilocybe serbica* var. *arcana*	54	18	0.000–0.024	0.000–2.237	0.000–0.794	0.412–7.922	0.002–8.878
*Psilocybe serbica* var. *bohemica*	20	8	0.008–0.093	0.234–2.473	0.173–1.282	0.027–2.485	1.553–15.543
*Psilocybe serbica* var. *moravica*	6	1	0.053–0.249	0.293–0.822	0.453–2.012	0.061–0.386	5.655–14.158
*Psilocybe* sp.	6	1	―	0.282–0.965	0.031–0.144	1.489–2.051	0.512–1.892
*Psilocybe subaeruginosa*	2	1	―	0.009–0.011	<LOQ	0.081–0.326	0.102–0.195
*Psilocybe zapotecorum*	2	1	0.021	0.430–0.481	0.208–0.211	0.293–0.369	9.022–9.655
*Stropharia aeruginosa* *	3	1	<LOD	<LOD	<LOD	<LOD	<LOD
*Agaricus bisporus* *	20	1	<LOD	<LOD	<LOD	<LOD	<LOD

fb—number of analyzed fruiting bodies, n—number of individual collections, n.a.—not analyzed, LOD—limit of detection, * PS/PSB negative controls.

## Data Availability

Not applicable.

## Supplementary Materials

The following supporting information can be downloaded at: https://www.mdpi.com/article/10.3390/ijms232214068/s1.
