# Supplementary material for: Extensive Collection of Psychotropic Mushrooms with Determination of Their Tryptamine Alkaloids
Source: Int J Mol Sci. 2022 Nov 15;23(22):14068. doi: 10.3390/ijms232214068 (PMC9693126; doi:10.3390/ijms232214068)
Supplement: Supplementary file 1 [file ijms-23-14068-s001.zip › ijms-1921808-supplementary.pdf]

International Journal of Molecular Sciences

Supplementary Table S1. Data on analyzed mushroom collections.

ID	Mushroom species	Coll.Date*	Origin	Fungarium ID	GenBank	Analysis
PS-01	*Panaeolus foenisecii*	14.06.2015	Czech Republic	PRM 934273	n.a.	May 2016
PS-02	*Panaeolus cinctulus* cf.	24.05.2015	Italy	PRM 934316	MW352022	May 2016
PS-03	*Panaeolus cinctulus*	09.05.2015	Czech Republic	PRM 934317	n.a.	May 2016
PS-04	*Psilocybe subaeruginosa*	12.07.2015	Tasmania	PRM 934325	n.a.	May 2016
PS-05	*Panaeolus papilionaceus*	30.09.2015	Czech Republic	(PRM 934907)	n.a.	May 2016
PS-06	*Panaeolus olivaceus*	10.10.2015	Czech Republic	PRM 935914	MW352021	May 2016
PS-07	*Psilocybe medullosa*	10.10.2015	Czech Republic	PRM 934921	n.a.	May 2016
PS-08	*Inocybe calamistrata*	22.09.2015	Czech Republic	HR 97960	n.a.	May 2016
PS-09	*Psilocybe serbica* var. *bohemica*	08.11.2015	Czech Republic	(PRM 923899)	n.a.	May 2016
PS-10	*Psilocybe cyanescens*	08.11.2015	Czech Republic	(PRM 923257)	n.a.	May 2016
PS-11	*Psilocybe serbica* var. *arcana*	27.10.2015	Czech Republic	PRM 934861	n.a.	May 2016
PS-12	*Psilocybe serbica*	27.10.2015	Bosnia and Herzegovina	PRM 934969	n.a.	May 2016
PS-13	*Pluteus salicinus*	21.07.2012	Sweden	-	n.a.	May 2016
PS-14	*Psilocybe semilanceata*	26.09.2015	Sweden	PRM 935886	n.a.	May 2016
PS-15	*Panaeolus cinctulus*	27.08.2008	Sweden	PRM 935887	n.a.	May 2016
PS-16	*Psilocybe medullosa*	02.09.2011	Sweden	PRM 935889	n.a.	May 2016
PS-17	*Pholiotina cyanopus*	23.09.2015	Sweden	PRM 935888	n.a.	May 2016
PS-19	*Psilocybe medullosa*	08.11.2015	Czech Republic	PRM 934957	n.a.	May 2016
PS-20	*Psilocybe ovoideocystidiata*	01.12.2015	Maryland, USA	(PRM 934866)	n.a.	May 2016
PS-21	*Psilocybe* sp.	29.01.2016	Australia	PRM 951401	MN901951	May 2016
PS-23	*Psilocybe serbica* var. *bohemica*	05.11.2016	Czech Republic	-	n.a.	Sep. 2017
PS-26	*Psilocybe serbica* var. *moravica*	31.10.2016	Czech Republic	(PRM 860954)	n.a.	Sep. 2017
PS-30	*Psilocybe ovoideocystidiata*	25.05.2016	Virginia, USA	PRM 945683	n.a.	Sep. 2017
PS-31	*Panaeolina foenisecii*	08.06.2016	Czech Republic	-	n.a.	Sep. 2017
PS-33	*Inocybe corydalina*	18.08.2016	Czech Republic	HR 103893	n.a.	Sep. 2017
PS-42	*Pluteus glaucotinctus*	08.11.2015	Martinique	V. Antonín VA15.243	MN901948	Sep. 2017
PS-43	*Psilocybe fimetaria*	Nov. 2016	France	PRM 945733	n.a.	Sep. 2017
PS-44	*Inocybe aeruginascens*	8.6.2013	Hungary	B. Dima BD5076	n.a.	April 2018
PS-45	*Gymnopilus dilepis*	15.6.2017	Malaysia	PRM 946008	MN901949, MN900720	April 2018
PS-46	*Psilocybe fuscofulva*	19.9.2017	Czech Republic	PRM 945880	n.a.	April 2018
PS-47	*Psilocybe fuscofulva*	20.9.2017	Czech Republic	PRM 945859	n.a.	April 2018
PS-48	*Pluteus salicinus*	07.10.2017	Czech Republic	PRM 945836	n.a.	April 2018
PS-50	*Psilocybe caerulescens*	11.08.2016	Mexico	-	MN901952	April 2018
PS-51	*Psilocybe zapotecorum*	09.07.2016	Mexico	-	MN901953	April 2018
PS-52	*Psilocybe mexicana*	18.07.2016	Mexico	-	MN901954	April 2018
PS-53	*Pluteus americanus*	26.10.2017	Detroit, USA	PRM 946472	MN901950	April 2018
PS-54	*Pluteus americanus*	26.10.2017	Detroit, USA	(PRM 946472)	n.a.	April 2018
PS-55	*Psilocybe caerulipes*	08.09.2017	Detroit, USA	-	n.a.	April 2018
PS-56	*Pluteus americanus*	22.09.2017	Kentucky, USA	PRM 946474	n.a.	April 2018

PS-57	*Psilocybe caerulipes*	12.09.2017	Kentucky, USA	PRM 946475	n.a.	April 2018
PS-58	*Psilocybe ovoideocystidiata*	06.03.2017	Kentucky, USA	PRM 946476	n.a.	April 2018
PS-59	*Psilocybe semilanceata*	01.11.2018	Czech Republic	PRM 951339	n.a.	April 2019
PS-60	*Psilocybe fuscofulva*	26.09.2018	Czech Republic	PRM 951350	n.a.	April 2019
PS-63	*Psilocybe serbica* var. *bohemica*	17.11.2018	Czech Republic	-	n.a.	April 2019
PS-64	*Psilocybe serbica* var. *bohemica*	18.11.2018	Czech Republic	PRM 951316	n.a.	April 2019
PS-65	*Psilocybe fimetaria*	03.11.2018	Czech Republic	PRM 951396	MN901955, LR760712	April 2019
PS-66a	*Inocybe calamistrata*	06.10.2018	Czech Republic	PRM 952106	n.a.	April 2019
PS-66 b	*Inocybe calamistrata*	06.10.2018	Czech Republic	PRM 952106	n.a.	April 2019
PS-67	*Inocybe corydalina*	31.08.2010	Czech Republic	PRM 899248	n.a.	April 2019
PS-68	*Inocybe calamistrata*	10.10.2013	Czech Republic	PRM 923127	n.a.	April 2019
PS-69	*Inocybe calamistrata*	12.09.2013	Czech Republic	PRM 922987	n.a.	April 2019
PS-70	*Psilocybe serbica* var. *arcana*	14.11.2018	Germany	PRM 952111	n.a.	April 2019
L-05	*Psilocybe serbica* var. *arcana*	08.11.2015	Czech Republic	PRM 952706	n.a.	May 2016
L-06	*Psilocybe serbica* var. *arcana*	17.10.2015	Czech Republic	PRM 952707	n.a.	May 2016
L-07	*Psilocybe serbica* var. *arcana*	24.10.2015	Czech Republic	PRM 952708	n.a.	May 2016
L-08	*Psilocybe cyanescens*	31.10.2015	Czech Republic	PRM 952709	n.a.	May 2016
L-09	*Psilocybe cubensis*	March 2016	cultivation	-	n.a.	May 2016
L-10	*Psilocybe semilanceata*	26.09.2015	Czech Republic	PRM 952710	n.a.	May 2016
L-12	*Psilocybe cubensis*	April 2014	cultivation	PRM 952711	n.a.	May 2016
L-13	*Psilocybe serbica* var. *arcana*	May 2014	Czech Republic	-	n.a.	May 2016
H-01	*Psilocybe cyanescens*	21.10.2016	Czech Republic	PRM 952704	n.a.	Sep. 2017
H-02	*Psilocybe cyanescens*	26.10.2016	Czech Republic	PRM 952704	n.a.	Sep. 2017
H-03	*Psilocybe serbica* var. *arcana*	26.10.2016	Czech Republic	PRM 952691	n.a.	Sep. 2017
H-04	*Psilocybe serbica* var. *arcana*	27.10.2016	Czech Republic	PRM 934295	n.a.	Sep. 2017
H-05	*Psilocybe serbica* var. *arcana*	28.10.2016	Czech Republic	PRM 952692	n.a.	Sep. 2017
H-06	*Psilocybe serbica* var. *arcana*	28.10.2016	Czech Republic	PRM 952693	n.a.	Sep. 2017
H-07	*Psilocybe serbica* var. *arcana*	28.10.2016	Czech Republic	PRM 952694	n.a.	Sep. 2017
H-08	*Psilocybe serbica* var. *arcana*	28.10.2016	Czech Republic	PRM 952695	n.a.	Sep. 2017
H-09	*Psilocybe serbica* var. *arcana*	30.10.2016	Czech Republic	PRM 952696	n.a.	Sep. 2017
H-10	*Psilocybe serbica* var. *arcana*	04.11.2016	Czech Republic	PRM 952705	n.a.	Sep. 2017
H-11	*Psilocybe serbica* var. *arcana*	04.11.2016	Czech Republic	PRM 952705	n.a.	Sep. 2017
H-12	*Psilocybe serbica* var. *bohemica*	23.12.2016	Czech Republic	PRM 946005	n.a.	Sep. 2017
H-13	*Psilocybe semilanceata*	16.09.2016	Czech Republic	PRM 952697	n.a.	April 2018
H-14	*Psilocybe semilanceata*	30.09.2016	Czech Republic	PRM 952698	n.a.	April 2018
H-15	*Psilocybe serbica* var. *arcana*	28.10.2016	Czech Republic	PRM 952699	n.a.	April 2018
H-16	*Psilocybe serbica* var. *arcana*	04.11.2016	Czech Republic	PRM 952700	n.a.	April 2018
H-17	*Psilocybe serbica* var. *arcana*	04.11.2016	Czech Republic	PRM 952701	n.a.	April 2018
H-18	*Psilocybe serbica* var. *bohemica*	04.11.2016	Czech Republic	PRM 952702	n.a.	April 2018
H-19	*Psilocybe medullosa*	04.11.2016	Czech Republic	PRM 952703	n.a.	April 2018
H-20	*Psilocybe cubensis*	27.01.2017	cultivation	-	n.a.	April 2018

H-21	*Psilocybe cubensis*	24.02.2017	cultivation	-	n.a.	April 2018
H-22	*Psilocybe serbica* var. *bohemica*	05.12.2017	Czech Republic	-	n.a.	April 2018
H-23	*Psilocybe serbica* var. *bohemica*	06.12.2017	Czech Republic	-	n.a.	April 2018
Negative control						
-	*Stropharia aeruginosa*	25.10.2016	Czech Republic	PRM 945622	n.a.	throu-ghout

*day.month.year

Supplementary Table S2. Scientific names of mushroom species investigated in this study.

Mushroom species (according to Index Fungorum*)	Family	Important synonyms
Agaricus bisporus (J.E. Lange) Imbach, Mitt. naturf. Ges. Luzern 15: 15 (1946)	*Agaricaceae*	
Gymnopilus dilepis (Berk. & Broome) Singer, Lilloa 22: 560 (1951) [1949]	*Strophariaceae*	
Inocybe aeruginascens Babos, Fragm. Bot. Mus. Hist.-Nat. Hung. 6(1-6): 21 (1968)	*Crepidotaceae*	
Inocybe calamistrata (Fr.) Gillet, Hyménomycètes (Alençon): 513 (1876) [1878]	*Crepidotaceae*	*Inosperma calamistratum* (Fr.) Matheny & Esteve-Rav.
Inocybe corydalina Quél., Mém. Soc. Émul. Montbéliard, Sér. 2 5: 543 (1875)	*Crepidotaceae*	
Panaeolina foenisecii (Pers.) Maire, Treb. Mus. Ciènc. nat. Barcelona, sér. bot. 15(no. 2): 109 (1933)	*Strophariaceae*	
Panaeolus cinctulus (Bolton) Sacc., Syll. fung. (Abellini) 5: 1124 (1887)	*Strophariaceae*	*Panaeolus subbalteatus* (Berk. & Broome) Sacc.
Panaeolus olivaceus F.H. Møller, Fungi of the Faeröes, Part I: Basidiomyceten: 171 (1945)	*Strophariaceae*	
Panaeolus papilionaceus (Bull.) Quél., Mém. Soc. Émul. Montbéliard, Sér. 2 5: 152 [122 repr.] (1872)	*Strophariaceae*	
Pholiotina cyanopus (G.F. Atk.) Singer, Trudy Bot. Inst. Akad. Nauk SSSR, ser. 2, Sporov. Rast. 6: 425 (1950)	*Bolbitiaceae*	*Conocybe cyanopus* (G.F. Atk.) Kühner
Pluteus americanus (P. Banerjee & Sundb.) Justo, E.F. Malysheva & Minnis, in Justo, Malysheva, Bulyonkova, Vellinga, Cobian, Nguyen, Minnis & Hibbett, Phytotaxa 180(1): 62 (2014)	*Pluteaceae*	
Pluteus glaucotinctus E. Horak, Bull. Jard. Bot. natn. Belg. 47(1-2): 88 (1977)	*Pluteaceae*	
Pluteus salicinus (Pers.) P. Kumm., Führ. Pilzk. (Zerbst): 99 (1871)	*Pluteaceae*	
Psilocybe caerulescens Murrill, Mycologia 15(1): 20 (1923)	*Strophariaceae*	*Psilocybe weilii* Guzmán, Stamets & F. Tapia, *Psilocybe mazatecorum* R. Heim
Psilocybe caerulipes (Peck) Sacc., Syll. fung. (Abellini) 5: 1051 (1887)	*Strophariaceae*	
Psilocybe tasmaniana Guzmán & Watling, Notes R. bot. Gdn Edinb. 36(1): 207 (1978)	*Strophariaceae*	
Psilocybe cubensis (Earle) Singer, Sydowia 2(1-6): 37 (1948)	*Strophariaceae*	*Stropharia cubensis* Earle
Psilocybe cyanescens Wakef., Trans. Br. mycol. Soc. 29(3): 141 (1946)	*Strophariaceae*	
Psilocybe fimetaria (P.D. Orton) Watling, Lloydia 30: 150 (1967)	*Strophariaceae*	
Psilocybe fuscofulva Peck, Bull. N.Y. St. Mus. nat. Hist. 1(no. 2): 7 (1887)	*Strophariaceae*	*Psilocybe atrobrunnea* (Lasch) Gillet sensu Guzmán, sensu auct.; *Psilocybe turficola* Favre nom. inval.
Psilocybe medullosa (Bres.) Borovička, C.C.H. 84(4): 114 (2007)	*Strophariaceae*	*Phaeogalera medullosa* (Bres.) M.M. Moser, *Psilocybe tenax* s. auct.

Psilocybe mexicana R. Heim, Revue Mycol., Paris 22(1): 77 (1957)	*Strophariaceae*	
Psilocybe ovoideocystidiata Guzmán & Gaines, International Journal of Medicinal Mushrooms (Redding) 9(1): 75 (2007)	*Strophariaceae*	
Psilocybe semilanceata (Fr.) P. Kumm., Führ. Pilzk. (Zerbst): 71 (1871)	*Strophariaceae*	
Psilocybe serbica M.M. Moser & E. Horak, Z. Pilzk. 34(3-4): 138 (1969) [1968]	*Strophariaceae*	
Psilocybe serbica var. *arcana* (Borov. & Hlaváček) Borov., Oborník & Noordel., in Borovička, Noordeloos, Gryndler & Oborník, Mycol. Progr. 10(2): 153 (2011)	*Strophariaceae*	*Psilocybe arcana* Borovička & Hlaváček
Psilocybe serbica var. *bohemica* (Šebek) Borov., Oborník & Noordel., in Borovička, Noordeloos, Gryndler & Oborník, Mycol. Progr. 10(2): 153 (2011)	*Strophariaceae*	*Psilocybe bohemica* Šebek ex Šebek
Psilocybe serbica var. *moravica* (Borov.) Borov., Oborník & Noordel., in Borovička, Noordeloos, Gryndler & Oborník, Mycol. Progr. 10(2): 153 (2011)	*Strophariaceae*	*Psilocybe moravica* Borovička
Psilocybe subaeruginosa Cleland, Trans. Roy. Soc. S. Australia 51: 305 (1927)	*Strophariaceae*	
Psilocybe zapotecorum R. Heim, Revue Mycol., Paris 22(1): 77 (1957)	*Strophariaceae*	
Stropharia aeruginosa (Curtis) Quél., Mém. Soc. Émul. Montbéliard, Sér. 2 5: 141 (1872)	*Strophariaceae*	

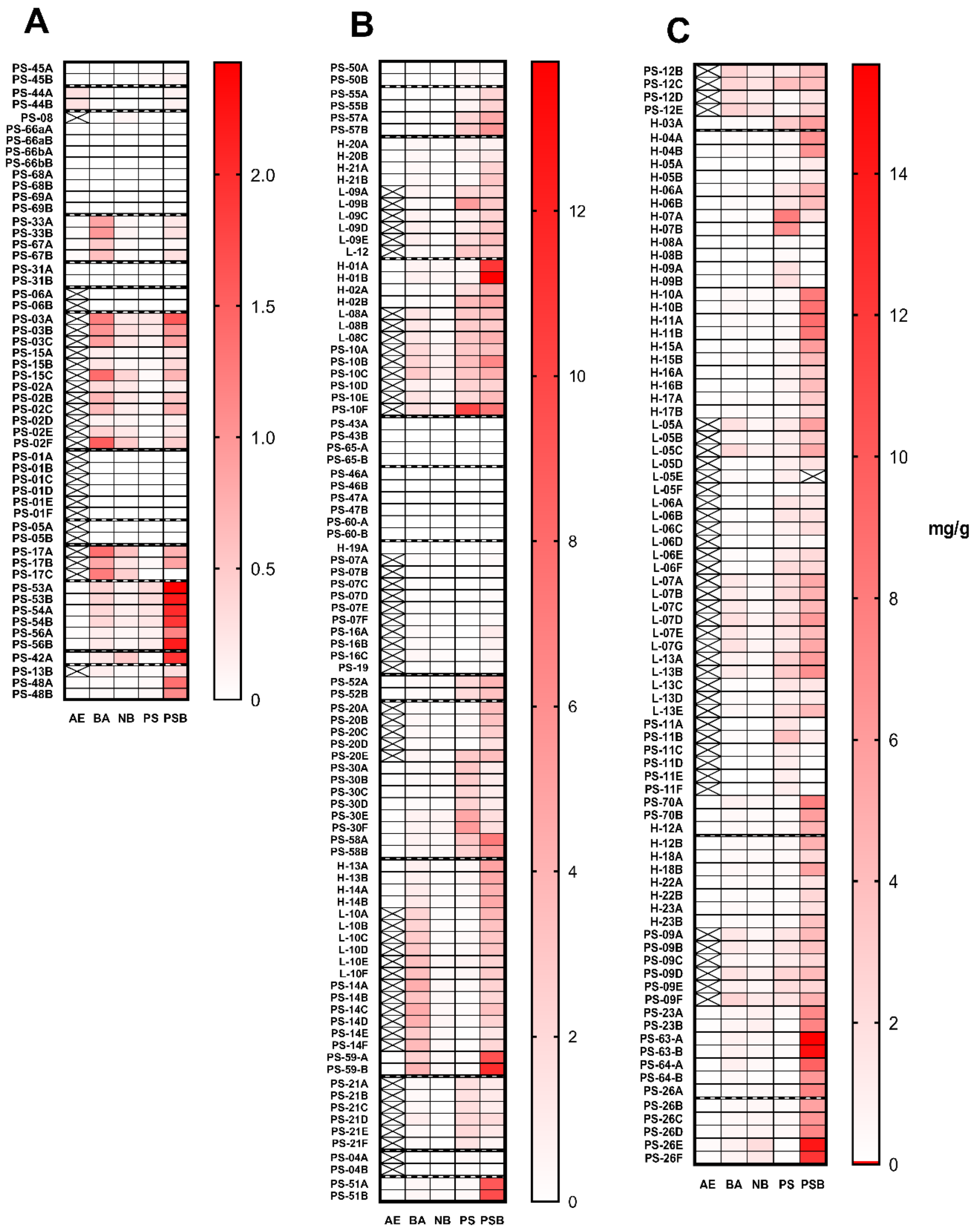

Supplementary Figure S1. Tryptamine concentrations in analyzed mushrooms (mg/g in dry mass) presented in heatmaps. Mushroom species are separated by dashed lines. (**A**) Non-*Psilocybe* genera. (**B**) *Psilocybe* species excluding *P. sebica* complex. (**C**) *P. serbica* complex.
